# Generative models of birdsong learning link circadian fluctuations in song variability to changes in performance

**DOI:** 10.1371/journal.pcbi.1011051

**Published:** 2023-05-01

**Authors:** Samuel Brudner, John Pearson, Richard Mooney

**Affiliations:** 1 Department of Neurobiology, Duke University School of Medicine, Durham, North Carolina, United States of America; 2 Department of Biostatistics & Bioinformatics, Duke University, Durham, North Carolina, United States of America; Tilburg University Faculty Humanities: Tilburg University Tilburg School of Humanities and Digital Sciences, NETHERLANDS

## Abstract

Learning skilled behaviors requires intensive practice over days, months, or years. Behavioral hallmarks of practice include exploratory variation and long-term improvements, both of which can be impacted by circadian processes. During weeks of vocal practice, the juvenile male zebra finch transforms highly variable and simple song into a stable and precise copy of an adult tutor’s complex song. Song variability and performance in juvenile finches also exhibit circadian structure that could influence this long-term learning process. In fact, one influential study reported juvenile song regresses towards immature performance overnight, while another suggested a more complex pattern of overnight change. However, neither of these studies thoroughly examined how circadian patterns of variability may structure the production of more or less mature songs. Here we relate the circadian dynamics of song maturation to circadian patterns of song variation, leveraging a combination of data-driven approaches. In particular we analyze juvenile singing in learned feature space that supports both data-driven measures of song maturity and generative developmental models of song production. These models reveal that circadian fluctuations in variability lead to especially regressive morning variants even without overall overnight regression, and highlight the utility of data-driven generative models for untangling these contributions.

## Author summary

Children learn many critical behaviors like language in part by imitating adults. For young learners, effective practice is thought to require random variation that can produce unexpectedly successful–or unsuccessful–outcomes. This kind of practice lasts weeks or months, so it is also structured by the learner’s circadian rhythm. In this paper we analyze the song of juvenile male zebra finches, which also learn to imitate adult vocalizations through extensive practice. We validate and deploy a novel combination of analysis tools to study this behavior, allowing us to relate vocal variation and performance using a succinct set of vocal features learned directly from song recordings. Specifically, we investigate how overnight changes in song variability affects performance quality. Although we do not find evidence that average song performance gets better or worse overnight–a subject of debate in the field–we show nonetheless that heightened morning variability reintroduces immature performance variations that were avoided the evening before. Thus, heightened morning variability may allow birds to avoid overcommitting to recently learned solutions. Our approach may be broadly useful to researchers interested in the connections between vocal variation and performance in this model of juvenile imitative learning.

## Introduction

Children learn many skills, including speech, music, and athletics, by imitating the expert behaviors of adults. Often this kind of learning depends on weeks or months of practice. Though performance improves overall during this extended period of practice, it also depends on acute exploratory variation in behavior, as well as processes operating at intermediate—for example, circadian—timescales. Therefore, a major challenge is to understand how patterns of performance arise from acute exploratory variation and circadian processes as well as from systematically learned changes. Here we address this challenge by applying data-driven methods to quantify behavior during a complex, multi-week form of juvenile imitative motor learning.

Juvenile male zebra finches memorize and vocally copy the temporally precise and complex polysyllabic song motif of an adult male tutor [1], affording the potential to understand how rendition-to-rendition variation during practice relates to behavioral learning. Notably, to accurately copy a tutor song, the juvenile engages in a form of sensorimotor learning that depends on auditory evaluations of its performance over tens or even hundreds of thousands of song renditions [2,3]. High levels of rendition-to-rendition variability, which are a hallmark of juvenile songs, are thought to promote vocal exploration important to learning [4–9]. Moreover, prior studies have detected circadian fluctuations in juvenile song performance [10–13], that in at least one case ultimately correlate with song learning outcomes [11].

Understanding how juvenile practice relates to song learning has been a longstanding goal of behavioral researchers. Classical studies sampled only a small fraction of song renditions generated over sensorimotor learning and compared sample spectrograms only qualitatively [14,15] or with linear cross-correlation methods that do not account for non-linear relationships between spectrogram pixels [16]. To study the entire corpus of songs generated during sensorimotor learning and account for non-linear spectrogram correlations, experimenter-predetermined acoustic features are now widely used [10,11,17–19], but preselected features may fail to capture complex spectral patterns in the data. In addition, procedures to summarize the within-syllable trajectories of these features often discard within-syllable temporal structure [17]. Recently, to address these limitations, researchers have applied non-linear, data-driven approaches to song learning datasets [12], although the novelty and complex behavior of these methods can make it hard to understand what factors affect their output.

Indeed, studies employing different analysis approaches have provided partially conflicting accounts of song learning in zebra finches. One influential study of select acoustic features concluded that learned changes to juvenile song accrue with practice each day but largely regress overnight, perhaps to prevent the consolidation of “inappropriate” learning, as in simulated tempering optimization algorithms [11,20]. However, this study measured the spectrotemporal complexity of individual song syllables to assay learning, and although this metric often increases over development, it may not directly reflect learning-related changes to song. A more recent study used a non-linear nearest neighbor-based technique to capture spectrogram structure that changed slowly over development and likely reflects song learning [12]. While this more recent study found that the quality of the “worst” syllables regresses overnight, other quantiles of the performance distribution consolidated fully [12], in contrast to the earlier account [11].

Neither of these studies systematically investigated how patterns of rendition-to-rendition acoustic variation might influence patterns of song maturity. Such an account may be critical to understand circadian patterns of performance: one prior study indicates that the amount of rendition-to-rendition variation in juvenile song oscillates each day, with variability being higher in the morning and lower in the evening [10]. One possibility is that the more variable morning song includes more immature variants that generate the appearance of regression at immature performance quantiles. Alternatively, song could regress on average overnight, while more variable morning song could include mature renditions far from the regressive average, generating the appearance of consolidation. Since some axes of acoustic variation may align with learning-related change and other axes may be orthogonal, investigating the influence of acoustic variation on performance requires an account of how syllable rendition variation is structured in a multidimensional acoustic space. To satisfy this requirement, we combine several data-driven analysis tools to study how variation in acoustic space influences overnight changes in song maturity.

As a common basis for quantifying song maturity and acoustic variation, we applied variational autoencoders [21–23] to learn low-dimensional, generative song feature sets from syllables spectrograms, extending an approach that effectively describes adult song to study song learning [24,25]. Based on these latent features, we developed a data-driven metric of song maturity, and used it to confirm that song performance changes overnight in a complex pattern instead of a simple pattern of “regressing” [12]. We then trained generative models to recapitulate the time-evolving mean and covariance structure of juvenile syllable distributions in latent space. Consistent with a prior report on the variance of classical acoustic features [10], these models exhibit circadian fluctuations in entropy, or overall amount of rendition-to-rendition song variation. In our models, overnight ‘regression’ at immature quantiles of performance emerges from a fluctuating circadian pattern of rendition-to-rendition song variability. Simulations that selectively eliminate this circadian pattern of variability do not show evidence of overnight regression across quantiles. Nonetheless, expanded morning variability in song performance may enable the juvenile zebra finch to avoid “overcommitting” to a local minimum it discovered the previous day, much in the same way that overnight regression has been proposed to benefit learning [11].

## Results

Our overarching goal was to quantify the acoustic changes that occur during sensorimotor learning in juvenile male zebra finches using a non-linear, data-driven approach. We focused on analyzing and quantifying changes at the level of individual syllables, a fundamental unit of song copying [11,12,26–28]. To investigate how overnight changes in song maturity arise from underlying patterns in song acoustic distributions, we used VAE-based syllable feature spaces [21–25], which provided a common basis for evaluating syllable maturity and for describing syllable acoustic distributions. We then developed a method using VAE coordinates to quantify the relative maturity of individual syllable renditions and characterize how maturity values change from one day to the next. Lastly, we modeled syllable rendition distributions in VAE latent space, simulated vocal development by sampling these distributions, and analyzed our simulations using our latent space-based maturity score. Together, these tools enabled us to understand how rendition-to-rendition variation contributes to overnight changes in syllable maturity.

### VAE latent representations of syllables produced throughout sensorimotor learning

**Fig 1:**
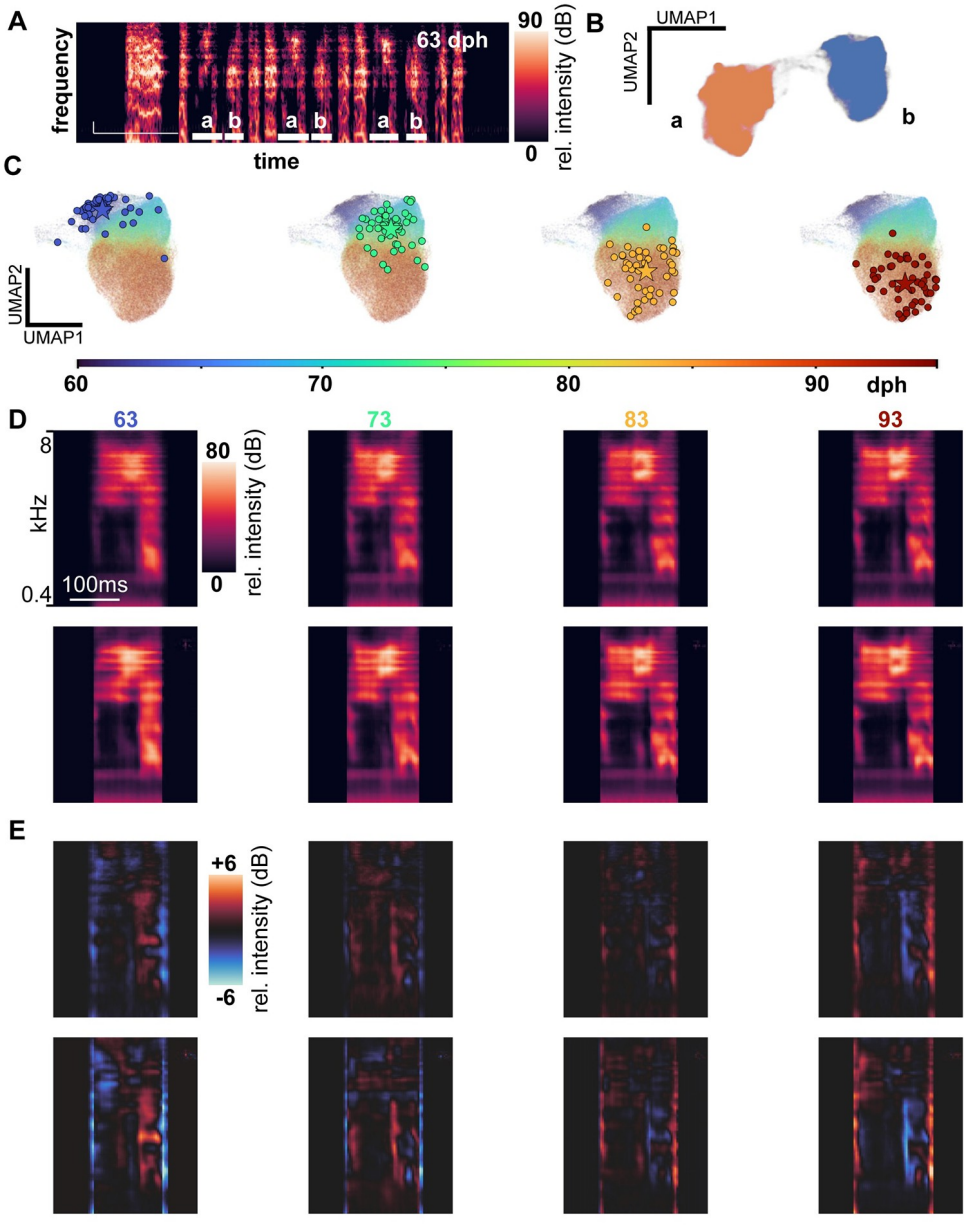
VAE representation of syllable development. **A**. An example plastic song motif containing two complex syllables, a and b. **B**. UMAP embedding of all complex syllable renditions produced by the bird with an example motif in **A**, colored by labeled syllable type. **C**. UMAP of all rendition latents, color coded by age at production time. The sampled renditions used to create average spectrograms in D-E are depicted as circles. The average location in latent space (“latent location” used to as decoder input in D-E) is represented by a star at average UMAP location. **D**. Average of spectrograms produced at 4 different ages in development (top; average of spectrograms with latent location given by circles in C), and decoder output at the mean latent space location at these ages (bottom; in C, star location at mean of UMAP coordinates visually represents mean latent coordinates). **E.** Contrasts depict the difference between average spectrograms at each age, and the average spectrogram across ages (top); or contrasts depict the difference between the decoded spectrogram at each age-specific, latent mean location and the cross-age average of these decoded images (bottom).

By the middle stages of sensorimotor learning (~60 days post hatch (dph)), zebra finch song mostly consists of acoustically clustered syllable types (example juvenile song motifs and component syllables depicted in Fig 1A). We sought to describe how the renditions of individual syllable types change during development, so we collected audio recordings of vocal repertoires from 5 normally tutored, individually housed juvenile male zebra finches during the middle to late stages of learning (approximately 60 to 100dph, see S1 Table and Methods). We computed spectrograms from these audio waveforms and set sound amplitude thresholds to segment motifs into component syllables. Although these syllable spectrograms capture spectrotemporal structure in syllable renditions, their high dimensionality and non-linear rendition-to-rendition correlations make them unsuitable for quantitatively describing developmental changes in the distributions of syllable features. Such a quantitative description requires techniques that reduce these high-dimensional datasets.

A number of dimensionality reduction techniques exist, with t-SNE [29,30] and UMAP [31] among the most widely used. These representations can preserve neighbor identities but do not learn generative feature spaces [32]. We followed recent work using variational autoencoders (VAEs) to represent vocal spectrograms, including zebra finch syllables [21–25]. These algorithms learn a compressed representation that preserves a consistent geometry. We relied on a previously published architecture and training procedure for this use case, which uses a convolutional network to map syllable spectrogram images into a 32-dimensional “latent” space (details of syllable data preparation are provided methods) [25]. For each animal, we trained the autoencoder on sounds sampled randomly throughout the recorded period (30,000 segmented sounds sampled per animal). For visualization, but not quantitative analysis, we further embedded latent representations in two-dimensional UMAP embeddings of the VAE latent representation of the data (parameters in Methods). It is common for distinct syllable types to emerge through differentiation of a common “protosyllable” produced early in development [17]. In order to facilitate analysis of syllable type distributions, we focused on song produced after 60 dph, when song syllable types form clusters in this embedding that are separate from one another and from other sounds. Thus, we could assign syllable labels and remove non-syllable sounds prior to downstream analysis (S1 Fig). Fig 1B depicts a UMAP embedding of the latent representations of all syllable renditions in an example finch. Separate clusters in the UMAP correspond to identifiable syllable types (i.e., syllables a and b; a sparse “bridge” of renditions between the clusters reflects the rare production of protosyllable-like renditions at ages near 60dph [17,33]). Because our encoder architecture had previously been applied only to adult syllables [25], we sought to evaluate whether the 32-dimensional latent space was sufficiently expressive to describe within-cluster variation in juvenile song. For individual juvenile syllables, we determined that at most 8 independent latent space dimensions were required to account for 99% of latent space variation, suggesting that the size of the latent space did not itself limit the representational capacity of the autoencoder on our dataset.

Coloring each rendition of a syllable as a function of the age at which it was produced revealed a systematic age-dependent shift within each syllable cluster (Fig 1C). To motivate subsequent quantitative analyses, we qualitatively assessed whether developmental trajectories of syllable position through latent space reflected systematic change in syllable spectrograms. Thus, to visualize systematic syllable spectrogram change, we sampled renditions from each syllable cluster at 10-day intervals in our recordings (example syllable in Fig 1C, 50 renditions/day depicted as circles; other syllables in S2 Fig) and generated within-day average spectrograms from the audio signal on these renditions (Fig 1D top row). We then calculated the within-day average location of these renditions in latent space (schematically represented as the mean of the UMAP locations of these points in Fig 1C, star), and decoded these average positions in latent space to generate synthetic spectrogram representations of these mean positions (Fig 1D, bottom row). To highlight changing structure, we subtracted an across-day spectrogram average from each within-day average spectrogram (Fig 1E, top row). Similarly, we subtracted the average of the decoded images from each age-specific decoded image (Fig 1E, bottom row). Visual inspection showed a close correspondence between the changes in real spectrogram averages and the decoded latent mean spectrograms. These observations indicate that developmental changes charted in VAE latent space can capture developmental changes to syllable spectrograms, and motivated further quantification.

### Quantifying syllable rendition maturity during sensorimotor learning

The observation of developmentally relevant structure in VAE latent space suggests that the space may support exploring complex relationships between the changing mean, variation, and maturity of syllable distributions. We reasoned that such an exploration would benefit from a direct, quantitative map from latent space—which encodes but does not *evaluate* syllable acoustic structure—to a measure of syllable maturity during learning. Such a measure would permit us, for example, to interpret the maturity of syllables simulated using developmental models based in VAE latent space.

**Fig 2:**
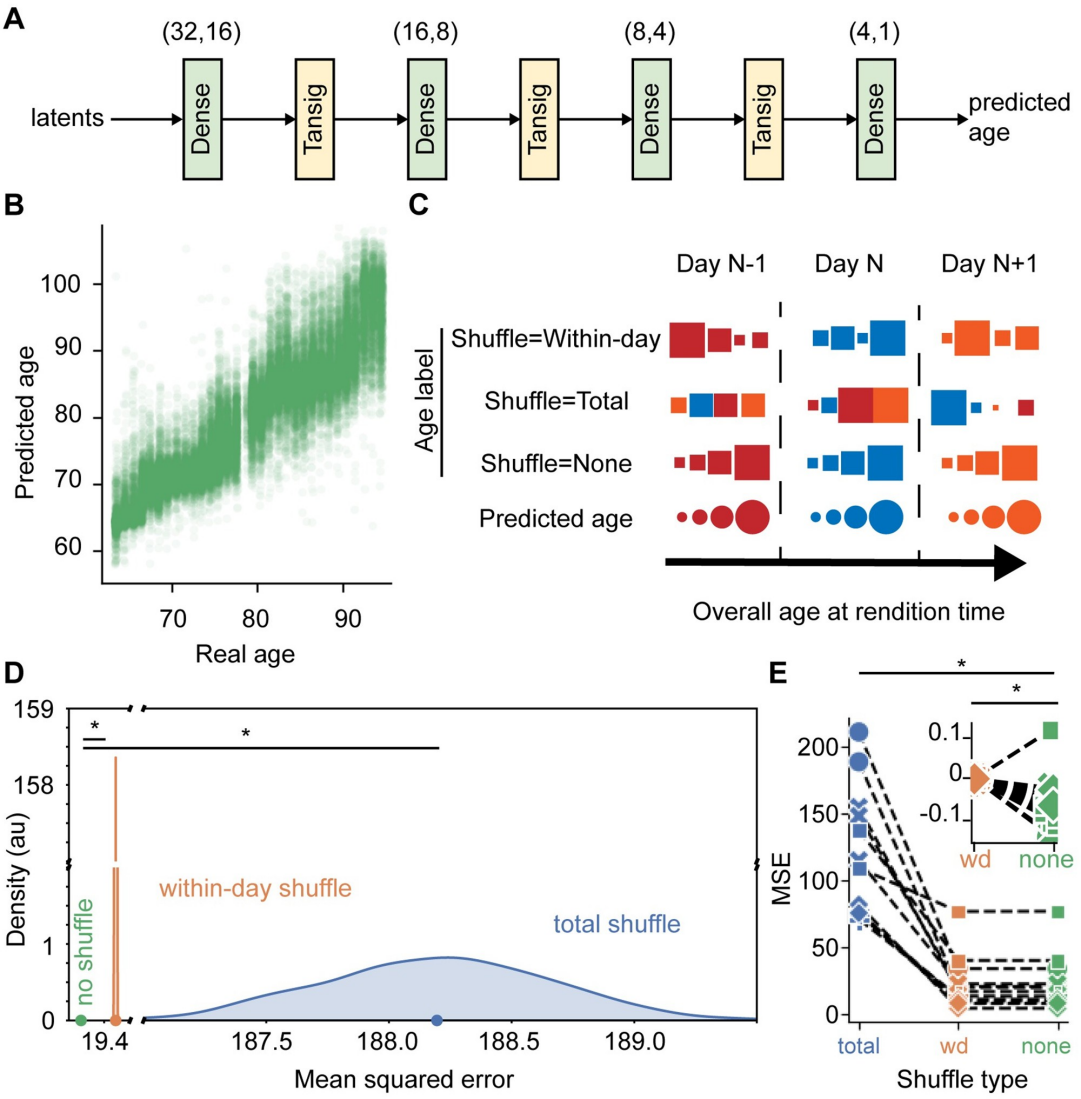
Latent representations encode short timescale changes in performance. **A**. Neural network design for predicting age. “Dense” layers are fully-connected linear layers with bias terms. “Tansig” layers implement the hyperbolic tangent sigmoid transfer function. Parenthetical number pairs indicate layer input and output size. **B**. Predicted age increases with the age at production time of test syllables held out from model training. **C**. Schematic of shuffle conditions. Each column of shapes corresponds to a test sound, which is assigned a predicted age, and a veridical (shuffle = none) or shuffled age label. **D**. Mean squared error of age predictions in days (green). Distribution and mean of MSE over 1000 random shuffles of age across all data in the test set (blue) or only within days (orange). All values were based on permutations of the example syllable data in **B**. Unshuffled performance exceeds 95% of the within-day shuffle permutations, with bootstrap significance indicated by star. In this case, unshuffled performance exceeds all within-day shuffles. **E**. Means of MSE for 1000 total (blue) or within-day (orange) permutation tests for every syllable, along with true MSE on held out data (green). Inset shows `dph shuffle’ and `none’ values with `dph shuffle’ value subtracted to emphasize that for all syllables but one, within-day shuffling increases MSE. Stars represent significance at Bonferroni-corrected alpha, as reported in text. Lines connect observations for individual syllables, while marker style indicates bird identity.

To satisfy this aim, we sought to estimate the conditional mean of the age distribution for each location in latent space. In preliminary analyses we observed that developmental trajectories in latent space were nonlinear, motivating us to seek nonlinear predictive methods (comparison to linear prediction in S3B Fig). We trained a feedforward neural network to predict bird age as a function of latent space location, by minimizing the squared error loss between actual and predicted ages for each embedded vocalization (Fig 2A and 2B; 705 trained parameters; our design was heuristically motivated as a simple pyramid architecture; we achieved similar results using a shallower model, S3A and S3B Fig, indicating performance did not depend sensitively on architecture). Trained networks predicted the age of held out test data better than chance (assessed by total shuffle control, Fig 2C-E). In addition, for 12 of 13 syllables examined, age prediction models based on VAE latents outperformed similar models that used spectrogram principal component representations matched for effective dimensionality (S3C Fig).

Since we ultimately sought to investigate circadian patterns in singing, we assessed whether our maturity scores capture short timescale (<24hrs) changes in performance. If so, model performance should be degraded by shuffling procedures that selectively break short-time scale relationships. For each syllable, we measured MSE after each of 1000 within-day shuffles (see shuffling procedure in Fig 2C; example distribution of MSE following 1000 within-day shuffles in Fig 2D). Models achieved lower MSE than 95% of within-day shuffled performance for 12 of 13 syllables. In an analysis across syllables, models outperformed the average score achieved after shuffling (Fig 2C and 2D; n = 13 syllables, F(2,24) = 58.208, p = 6.216*10^−10^; total shuffle—unshuffled MSE = 94.857, p = 1.6873*10^−5^, Tukey-Kramer; dph shuffle—unshuffled MSE = 0.060971, p = 0.016528, Tukey-Kramer; both significant at Bonferroni-corrected alpha = 0.025). Thus, this predicted age measure captures time trends in syllable renditions across both long (days to weeks) timescales and the short (within-day) timescales relevant to circadian processes.

### Quantifying changes in song maturity from one day to the next

**Fig 3:**
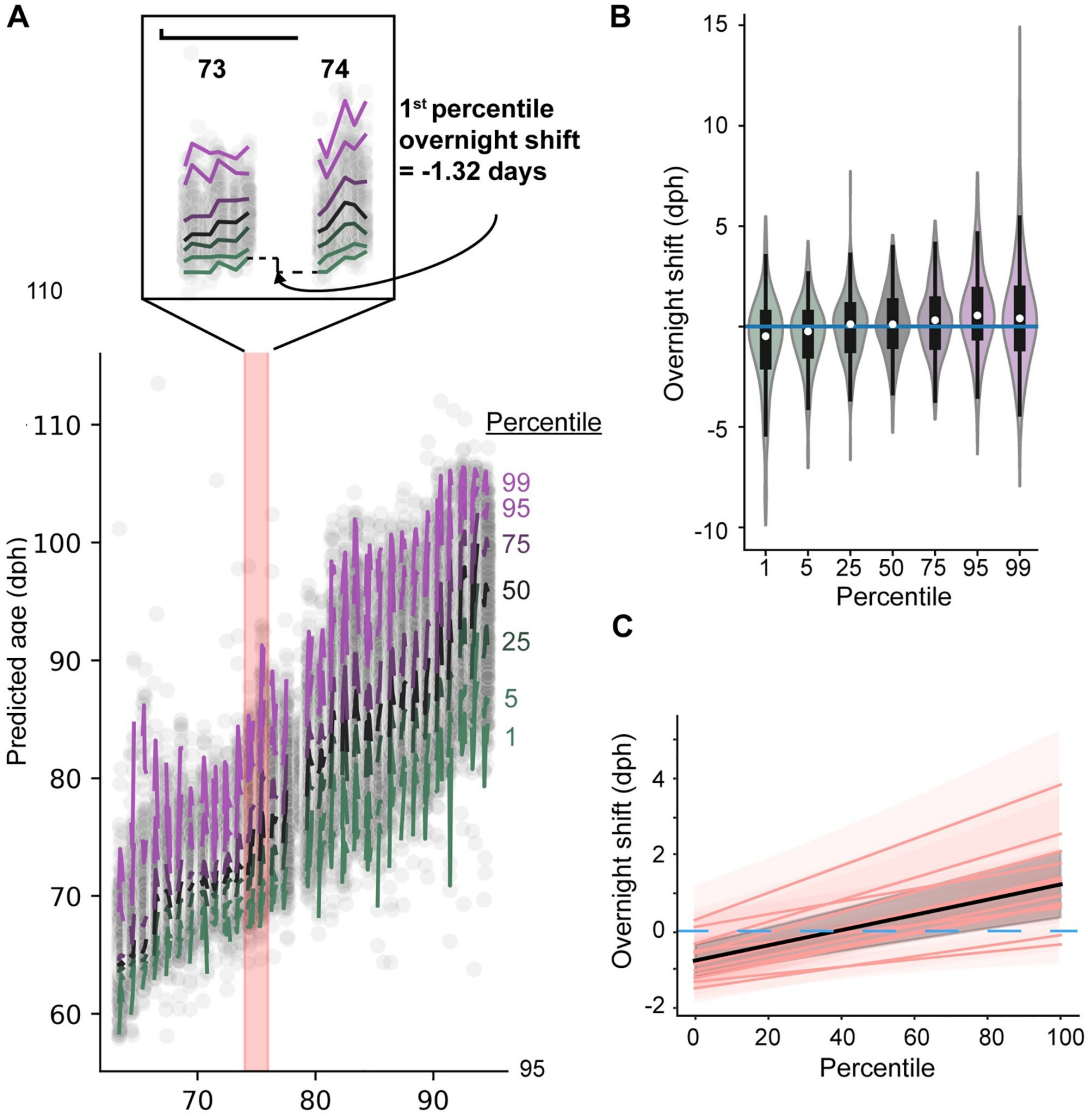
Overnight performance change depends on performance quantile. **A**. Predicted vs actual production age for held out renditions of an example syllable. Overlaid traces plot percentiles of the predicted age measure by production age for time-binned data. Inset shows two consecutive days, and the calculation of a percentile-wise overnight shift. **B**. Violin plot of percentile-wise overnight shifts for all overnight shifts in the dataset. **C**. Partial dependence of overnight shift value on percentile (expected value and 95% confidence interval depicted as black line and shaded region), marginalized over syllable and bird random effects. The fixed intercept and slope with percentile are significant, as reported in the text. Predictions for individual syllables conditioned on random effects at the bird and syllable level (pink).

Circadian processes generally, and sleep in particular, have been proposed to influence practice behavior and learning [10–13,34–38]. One prior study in juvenile zebra finches concluded that song matures each day but “regresses” overnight [11]. However, a more recent study argued that diurnal song changes largely consolidate overnight, with regression limited to immature quantiles of the distribution of maturity values [12]. In light of this discrepancy, we sought to investigate overnight changes in maturity further—including, ultimately, whether identifiable patterns of syllable development in VAE space can explain overnight maturity changes. As a prerequisite to this analysis, we first sought to establish whether overnight changes measured in VAE space were consistent with overnight regression in maturity, either overall or for immature quantiles only. To examine how predicted age varies over time, we binned syllables by production time (2.4 hr bins) and calculated the predicted age at multiple quantile levels (7 levels shown in Fig 3A; similar results with other binning choices are presented in S2 Table). To assess whether any predicted age quantiles exhibited overnight “regression,” we calculated the change overnight (i.e., from the last time bin of a preceding day to the first time bin of a subsequent day) in predicted age at each quantile level (Fig 3A and 3B). Only immature data quantiles appeared to exhibit overnight regression, reflected in a negative shift in predicted age values at the 1st and 5th percentiles. In contrast, we observed the opposite pattern in the two uppermost data quantiles (95^th^ and 99^th^ percentiles), which exhibited positive (“progressive”) overnight shifts in predicted age values.

We quantified the linear dependence of these overnight maturity shifts on quantile level (n = 1425 quantile-level overnight contrasts), and included random effects components in the intercept and slope at the bird (n = 5) and syllable (n = 13) levels (see methods). The tendency towards overnight regression was significantly quantile-dependent (shift increases by 2.0008+/-0.33559 days as quantile ranges from 0 to 1; p = 3.132*10^−9^, MATLAB coefTest) and is pulled towards regressive shifts at the lower range of quantiles by a significant negative intercept (-0.77332+/-0.21505, p = 0.00033407, MATLAB coefTest). The joint impact of these model factors is depicted in Fig 3C, which depicts this linear relationship of fixed effects as well as the syllable-level model predictions incorporating random effects. This quantile-dependent regression is broadly consistent with recent observations [12], although the progressive changes in the uppermost quantiles of the syllable maturity distribution have not been previously reported. Notably, our predicted age result is inconsistent with a model in which the acoustic distribution of syllable renditions simply translates towards a target location during diurnal practice, then shifts precisely “backwards” at night. (Overnight regression depended on performance quantile in a similar way for other quantile binning choices; S2 Table.)

### Quantifying the structure of acoustic variation during sensorimotor learning

**Fig 4:**
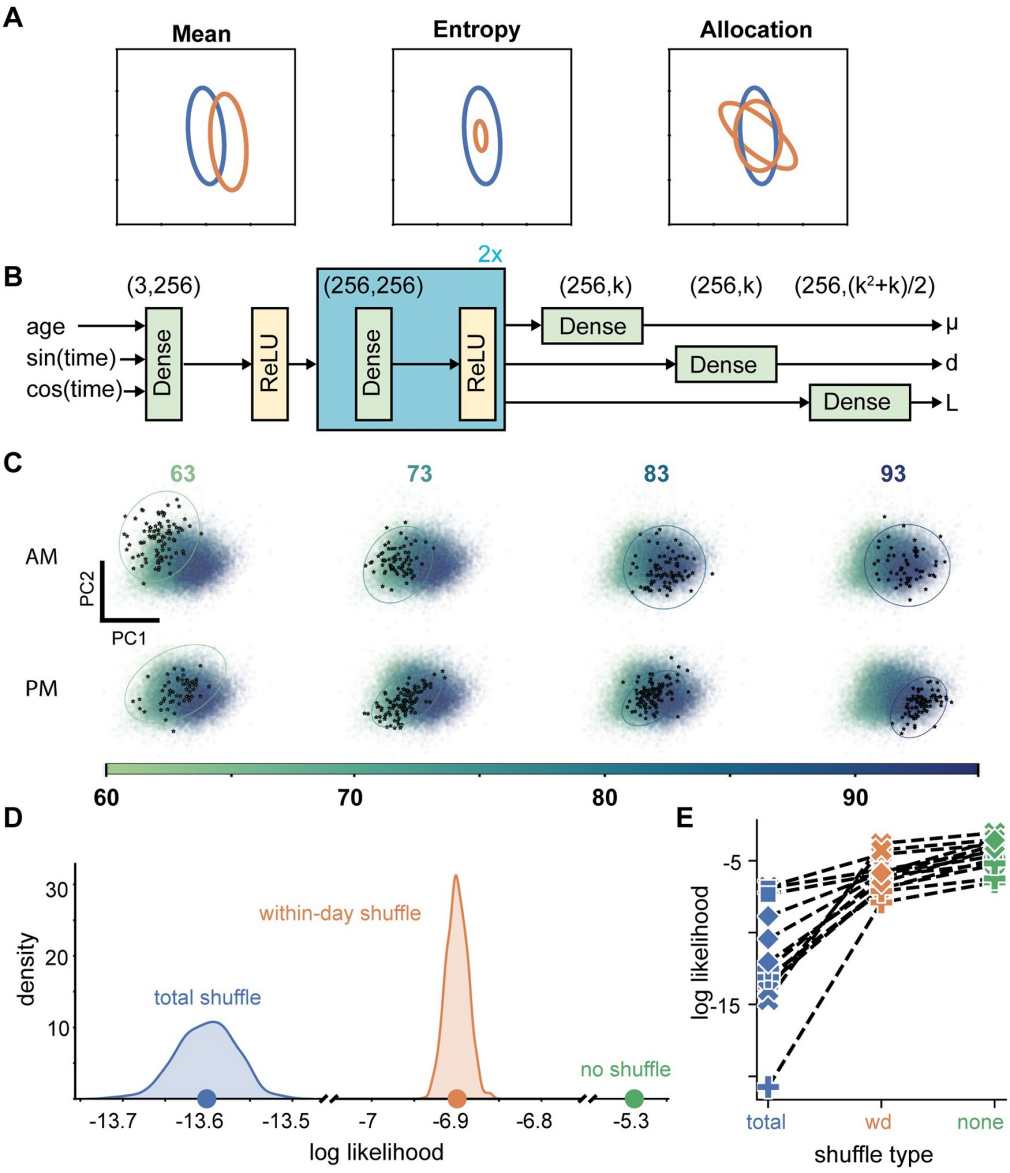
Gaussian models capture distributional changes in song at short timescales. **A**. A distribution’s mean and covariance structure can be represented as an ellipse, centered at the distribution mean, with axes oriented along the principal components of variation and axis magnitude determined by variance along principal components. Changes to mean and covariance reflect multiple factors that can each change independently: shifts of the distribution mean (left panel), changes in overall variation magnitude (middle panel), and changes that preserve entropy and mean location, but control the relative allocation of variation in different directions (right panel). **B**. Feedforward network architecture of developmental Gaussian models. “Dense” layers are fully-connected linear layers with bias terms. “ReLU” layers are rectified linear transfer function layers. Layers in the blue box were repeated. **C**. Example Gaussian model fits. Each panel emphasizes held out test renditions sung within a half-hour window occurring within 63dph morning (top left), 63dph evening (bottom left), etc. The Gaussian predicted at the center time of each half-hour window is depicted as a 95% confidence ellipse in its corresponding panel. Panel backgrounds contain the principal component projection of all example syllable renditions, colored by age at production time. **D**. Distribution of log likelihoods, calculated with respect a test set with totally shuffled production times (1000 permutations), within-day shuffled production times (1000 permutations), and unshuffled production times. Dots indicate distribution means. **E**. Mean log likelihood of shuffling experiments for all syllables. Lines connect values associated with individual syllables. Marker style indicates bird identity.

Although we can rule out the simplest deconsolidation model outlined above, more complex developmental models that involve overnight regression remain compatible with the predicted age patterns we observe. Ambiguity arises because predicted age may reflect not only translations of syllable distributions through latent space, but also may reflect these distributions’ rendition-to-rendition variation, which itself contains multiple kinds of structure. The absolute amount of variation is quantified as the entropy of the distribution of syllables. In addition, since syllables vary in multiple dimensions, variation may be allocated to varying degrees in different directions. For illustration, in Fig 4A, we depict a two-dimensional distribution as an ellipse, and independently manipulate its mean, the overall magnitude of the distribution’s variation (entropy), as well as the relative allocation of variation in different directions (allocation). Although the transformations of variation can affect the distribution of syllable maturity in complex ways, they do not change the mean in latent space; thus, they are distinct from the translations of the distributions of acoustic features that have typically been considered in connection to changes in maturity overnight [11,12].

We reasoned that a model of syllable production in latent space incorporating both mean syllable location and the basic variation features mentioned above would help us investigate the changes in maturity that occur overnight. In particular, we chose to model syllable production as a time-varying multivariate Gaussian distribution, the simplest (maximum entropy) model that captures the variation features mentioned above. One challenge to building these models is that syllable latent mean and variation parameters may change continuously at multiple timescales. Moreover, random sampling on any given day may obscure circadian structure of interest. To address these challenges, we modeled all syllable embeddings across the learning trajectory as arising from a multivariate Gaussian with a mean and covariance matrix that depend on age via a neural network. The neural networks were given production times as inputs, including an explicit circadian regressor—the sine and cosine of time of day—to facilitate identifying reliable circadian trends. The networks were trained to maximize the log likelihood of the observed latent embeddings. We were unsure of the complexity of this problem, and initially deployed an overparameterized model (Fig 4A; 141089 to 143402 trained parameters depending on k; see methods) because these often generalize well [39,40]. However, we subsequently also trained simpler models with 64 neurons per layer (10721 to 11956 trained parameters), or with reduced depth (75297 to 80180 trained parameters). These networks exhibited similar performance to our original model (S4 Fig).

Both across and within days, the Gaussian distributions calculated with the trained model reflect the changing location and spread of held out test data (example in Fig 4B). We quantified model performance on test data and again compared performance against the same data with the syllable rendition times randomly shuffled either across all ~30 days of sensorimotor learning or just within single days during this period (assessed via within-syllable repeated measures anova; n = 13 syllables; F(2,24) = 46.02; p = 6.1267*10^−9^). Model performance was profoundly disrupted when applied to shuffled datasets that disregard all production time information (no shuffle versus total shuffle, Fig 4C and 4D; no shuffle log likelihood—total shuffle log likelihood = 7.2945 +/- 0.97029; p = 1.9554*10^−5^, Tukey-Kramer). This disparity in performance indicates the reliance of the model on real age-related differences in syllable distributions. Within-day shuffling also impaired model performance (within-day shuffle, Fig 4C and 4D), albeit more subtly (no shuffle log likelihood—within-day shuffle log likelihood = 1.3925+/-0.17022; p = 8.3061*10^−6^, Tukey-Kramer), indicating that within-day model changes also can capture real, within-day changes in the distributions of syllable renditions. Thus, our time-varying latent Gaussian model provides a description of the evolving distribution of syllables during learning. In particular, this model is generative, and explicitly describes the changing mean and covariance of syllable distributions in latent space.

### Diurnal changes in variability explain changes in maturity from one day to the next

**Fig 5:**
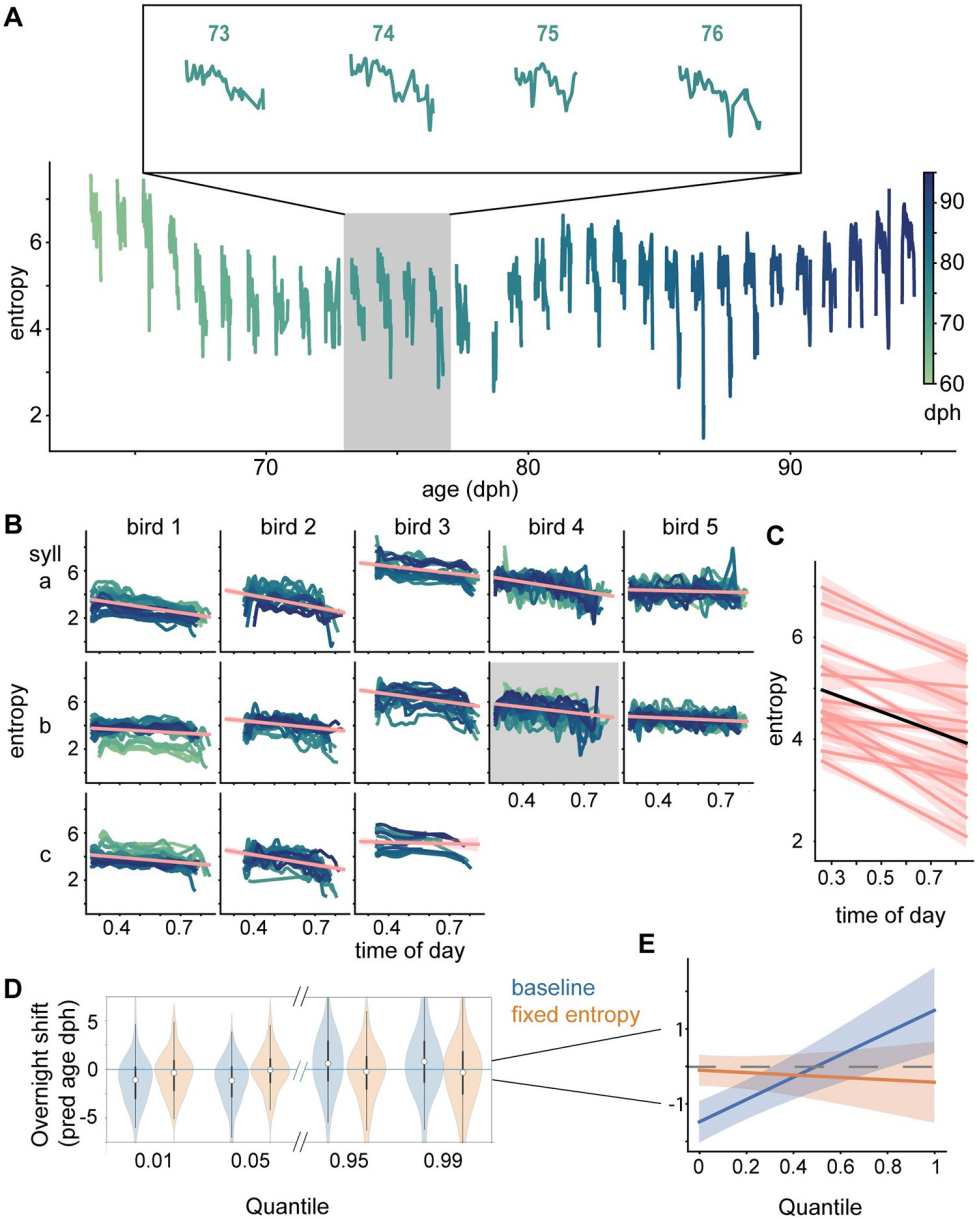
Circadian entropy fluctuations generate quantile-dependent overnight performance changes. **A**. Gaussian model entropy versus age for the example syllable. Inset depicts example days at higher temporal resolution. **B**. Within day changes of entropy for 5 birds (columns), separated by syllable type (rows). Shaded panel is fitted to the example syllable in A. **C**. Mixed effects model showing individual syllable trends (pink) and overall trend line (black). Entropy decreases with time of day (slope = -1.7586+/-0.29306, p = 1.9796*10^−9^) **D**. Overnight predicted age shifts in simulated data drawn from baseline Gaussian models or fixed entropy Gaussian models. **E**. Partial dependence of simulated overnight shifts on quantile and simulation entropy condition (baseline vs fixed). The effect of quantile (2.9618+/-0.60835 predicted age days, p = 1.1851*10^−6^, MATLAB coefTest) was offset by a significant interaction with entropy condition (quantile effect reduced by -3.2822+/-0.69456 in fixed entropy condition, p = 2.4062*10^−6^, MATLAB coefTest). A significant negative intercept (-1.4714+/-0.28112 days, p = 1.7766*10^−7^, MATLAB coefTest) primarily compensates for the spread of positive quantile effects; in the fixed entropy condition, this intercept is significantly reduced a main effect of simulation model (1.3729+/-0.26326 for “fixed entropy”, p = 1.9672*10^−7^, MATLAB coefTest).

To examine how changes in overnight predicted age depend on the structure of variation, we first sought to address whether entropy undergoes circadian changes by calculating the entropy of our models as a function of production age. Consistent with a report on rendition-to-rendition variability in physically defined acoustic features [10], we observed a daily pattern in the entropy of syllable distributions, with syllable distributions exhibiting relatively high entropy early in the day and relatively low entropy at the end of the day, precisely the pattern that would be required to explain the overnight changes in song maturity (Fig 5A and 5B). To quantitatively assess this pattern across multiple syllables and animals, we sampled our syllable-level models during periods of robust singing (>30 renditions of syllable type in surrounding 30 minutes; see methods). We calculated the entropy of these samples (n = 37728) and quantified the linear dependence of these values on time of day and included random effects components in the intercept and slope to account for the possibility that individual birds (n = 5) and syllables (n = 13) exhibit correlated deviations from the group average behavior. This model predicts an overall decrease in entropy over the day while successfully accounting for differences between syllables (pink lines, Fig 5B and 5C), (black line, Fig 5C; entropy decreases by 0.073275+/-0.0122/hr, p = 1.9795*10^−9^, MATLAB coefTest). In a secondary analysis that included a main effect of age and interaction between overall age and time of day (n = 37728 samples), as well as random effects at the bird (n = 5) and syllable (n = 13) level for all model coefficients, we observed that entropy decreases overall during development (by -0.032873+/- 0.015358 per day, p = 0.03233), and that the persistent circadian decrease in entropy (daily slope = -6.123+/-1.6616, p = 0.000228) also moderates with age (time-of-day by overall age interaction = 0.052082+/-0.019204 change in slope per day, p = 0.0066899). To ensure that non-linear VAE transformations did not introduce entropy trends that were absent in the distributions of spectrograms, we repeated these analyses on spectrogram principal component representations that were matched for the dimensionality of our VAE latent representations. Consistent with our models of development in VAE latent space, this analysis revealed a significant decrease in entropy each day (slope = -1.3662/day; p = 6.9*10^−5^) in a hierarchical linear regression (n = 32632 model samples) that included random effects at the bird (n = 5) and syllable (n = 13) levels. When we expanded the PC-based model to include fixed effects of overall age (dph) and an interaction between age and time-of-day, we observed an age-dependent effect: entropy decreased each day; this trend grew smaller later in development but was still negative at 95dph. Both the time-of-day effect (-5.46/day, p = 1.586*10^−5^) and time of day-by-age interaction (time-of-day slope grows by 0.05 day^-2^, p = 0.001) were significant. Thus, the circadian changes in VAE latent space entropy capture a phenomenon in juvenile song that is robust to song representation approaches.

Next, we sought to test directly whether the pattern of high morning entropy and low evening entropy could explain the quantile-dependence of overnight changes in predicted age. To address this question, we factored our model covariance matrices into separate terms representing independent entropy and variation allocation components. We used the Gaussian models we had trained previously as “baseline” models; these vary with respect to both variation factors to fit observed syllable distributions. From these baseline models, we derived “fixed entropy” Gaussian models that preserve the allocation factor from our baseline fits, but whose entropy is constrained to remain constant within each day (see methods). We then simulated syllable datasets by sampling from our baseline and fixed entropy models at timepoints corresponding to real syllable observations (see methods). We used the predicted age networks trained on real syllable observations to score our simulated observations, binned this measure into 7 quantiles, and calculated quantile-level overnight shifts in performance. The “baseline” simulation appeared to recapitulate the quantile-dependence of overnight predicted age shifts that we observed in real data, while overnight shifts in the “fixed entropy” simulation appeared similar across quantiles of the performance distribution (Fig 5D).

To quantify these relationships, we linearly regressed the quantile-level overnight shifts (n = 2870 shifts) against their quantile value, as well as a binary term indicating whether the shifts were simulated using a baseline or fixed-entropy model, and the interaction of these terms. We included random effects at the bird (n = 5) and syllable (n = 13) level for all the fixed effects coefficients. A significant effect of quantile was compensated by a significant interaction with model type, eliminating quantile-dependence in the fixed entropy simulation. Relatedly, the intercept depended significantly on model type, such that performance in the fixed entropy model did not regress by the large amounts observed at immature quantiles in baseline simulations; in fact, overnight shifts were not significantly different from zero at any quantile level when entropy was fixed within each day (Fig 5E). Thus heightened entropy each morning results in an expanded range of syllable rendition maturity scores compared to the prior evening, introducing regressive variants without a requirement for net overnight regression. We replicated these results with different quantile binning strategies (S4 Table) and in simulations using Gaussian models with different network architectures (S4 Table).

## Discussion

### Overview

Here we quantified juvenile zebra finch song behavior during sensorimotor learning using several complementary data-driven techniques. We used variational autoencoders [21–23] to learn a low-dimensional, highly informative acoustic space for characterizing juvenile song (following recent applications to adult song [24,25]). We used a deep predictive tool to score the acoustic maturity of every syllable rendition, as well as a deep generative model of age-varying syllable acoustic distributions. This model permitted us to analyze not only changes in latent syllable means but also features of the multidimensional rendition-to-rendition variation, separating the overall amount of variation from its relative allocation across multiple latent space directions. As we discuss below, this work builds on classical and more recent song analysis methods.

Although juvenile zebra finches produce better song copies with increasing reliability over many days and weeks of learning, singing is also regulated at shorter timescales. Most obviously, birds practice song during the day, and sleep at night. The interaction of processes operating at circadian timescales with long-term learning is poorly understood. In human skill learning, sleep leads to improved motor performance [34,35] and decreased susceptibility of recent motor learning to interference by subsequent experience [36]. Despite evidence of singing-like activity in song system nuclei during sleep [41], consistent with a theory that “replay” could consolidate or advance learning overnight, the behavioral effects of night sleep on singing quality remain a matter of debate. Most notably, Derégnaucourt et al. found that learned changes to song largely regress overnight [11]; while Kollmorgen et al, using a different approach, concluded that such regression only occurs in most immature syllable renditions, whereas other quantiles of the performance distribution underwent consolidation [12].

Consistent with Kollmorgen et al., we observed that the most immature margin of birds’ song repertoires regresses overnight, while the most mature margin appears to consolidate or even progress. We replicated this quantile-dependent regression in simulations based on the evolving distribution of song in VAE latent space, but not in simulations in which we selectively eliminated within-day changes in entropy. These “entropy-clamping” simulations did not exhibit significant overnight regression—nor progression—at any performance quantile, in contrast to Derégnaucourt et al. Thus, these simulations provide evidence that changes in entropy are sufficient to explain the complex, quantile-dependent pattern of overnight performance shifts observed by Kollmorgen et al. However, we note that high morning variability, including the production of highly deviant, immature variants, may benefit learning in the same way that overnight regression was proposed to benefit learning: juveniles may climb performance gradients in acoustic space as they practice each day, but can avoid overcommitting to a suboptimal local performance maximum by exploring far from recently learned local solution each morning.

### Novelty and relation to prior work

Our work complements studies that represent syllables in a low-dimensional space of physically defined acoustic features by reducing the need for experimenter choice during analysis. For example, Derégnaucourt et al. analyzed the within-syllable variation in Weiner entropy [11]. The authors interpret this feature as a measure of syllable acoustic complexity, itself a proxy of acoustic maturity, stemming from the observation that certain complex syllables have transitions between tonal and noisy elements that are slow in juveniles but fast in adults. Nevertheless, this interpretation requires excluding from analysis those syllables with persistently low entropy variance even after learning is complete. Thus, the conclusion in this study that song learning “regresses” overnight depends on the generalizability of observations of this particular feature and the syllables for which it was analyzed, as well as whether increased complexity specifically reflects the incorporation of learned complex structure.

Compared with Derégnaucourt et al. and other studies [10,11,17], our approach minimizes experimenter decisions about acoustic feature sets. Our feature sets also capture the sequencing of sounds within syllables, structure that is lost when within-syllable feature trajectories are summarized using the trajectory means and variances. As a tradeoff, the features we use do not correspond in a straightforward way to quantities with prior physical or acoustic definitions. Despite their abstract nature, the VAE latent features are interpretable as inputs to a generative model of realistic spectrograms, so in practice their meaning can be probed by observing how they influence the output of the spectrogram-generating decoder. Moreover, many outstanding behavioral and neural questions about song learning may not require features with simple physical interpretations. In fact, prior studies often do not leverage the physical interpretations of calculated features, but rather exploit those features’ post hoc correlations with age or other independent variables [11,42].

We formalize this age-correlation analysis with our predicted age score of syllable maturity (see Fig 2). Consistent with a recent study discussed below [11], but in contrast to entropy variance analysis [12], we do not observe simple “regression” of maturity overnight. Rather we see that only the least mature quantiles of the behavior regress overnight; other quantiles do not regress, and the most mature quantiles “progress.” These quantile-dependent overnight changes are echoed in another recent attempt to study song development using a data-driven maturity score. Kollmorgen et al. used a classifier of production day based on raw spectrograms in order to score syllable maturity [12]. This approach is conceptually similar to our neural network-based regression of production age against acoustic features to generate predicted age. Indeed, that study reported quantile-dependent changes in maturity that qualitatively agree with our observations. A key difference between the current study and Kollmorgen et al. is our use of a low-dimensional acoustic feature set (32 latent dimensions) as a basis for constructing maturity scores. This foundation enabled us to probe how the distribution of syllables in acoustic space influences patterns in maturity scores. As discussed further below, this technical innovation enabled us to identify the patterns of vocal production that underlie quantile-dependent shifts of maturity overnight.

Importantly, song is acoustically multidimensional, with the consequence that song could vary to different degrees along different axes. Our approach extends prior observations by capturing several aspects of the structure of rendition-to-rendition variation, including its entropy (a measure of variation magnitude) and relative allocation of this variation in different directions. We observed, consistent with a prior report [10], relatively high magnitude rendition-to-rendition acoustic variation in song produced in the morning compared with song produced in the evening. Through simulations, our Gaussian models enabled us to understand the underlying patterns that lead to quantile-dependence of overnight predicted age shifts. We tested the influence of circadian fluctuations in variation magnitude on the distribution of song rendition maturity. We conclude that without circadian fluctuations in variation magnitude, the other overnight changes in acoustic distributions—changes in mean location and changes in the relative allocation of variation along component directions—do not generate shifts in performance even at immature quantiles. In combination with the study by Kollmorgen [12], our results suggest reevaluating the observation of circadian and long-term changes to the spectrotemporal complexity measures quantified by Derégnaucourt et al [11]. These measures may reflect the ability to rapidly transition between different kinds of sounds, a capacity that may develop independently of the expression of specific, learned sound sequences.

### Broader implications and future directions

The variance of exploratory behavior, captured by entropy, plays a critical role in theories of reinforcement learning, where it scales the production of informative but potentially unrewarded behavior [6,9]. We see that high morning variation enables birds to produce progressive variants of greater maturity than they could produce the evening before. On the other hand, high morning variation leads to the production of more regressive variants as well. In this way, high morning variation preserves exploration in acoustic regions disfavored by recent learning, and may help birds avoid globally suboptimal, local performance maxima. These observations motivate future theoretical work on the impact of periodic fluctuations in variation magnitude on reinforcement learning. Moreover, our models provide a basis for future work describing how variation is allocated across acoustic dimensions; such allocation may influence or reflect ongoing learning.

Future experimental work that examines the mechanism of circadian variation patterns can help relate these behavioral patterns to learning processes. To this point, we note that a song-dedicated cortico-basal ganglia circuit (sCBG) is known to play an active role in shaping the variation of song output in juvenile zebra finches [4,43]. Moreover, song variability in adult finches is regulated in part by catecholamine levels [44,45] in the basal ganglia component of the sCBG. Therefore, circadian fluctuations in these catecholamines or other neuromodulators could lead to circadian changes in song variability. In addition, dysregulation of the transcription factor FoxP2 in song basal ganglia impairs the regulation of song variation by social context [46], while FoxP2 expression systematically falls during the morning hours of juvenile singing [19]. The analysis framework we develop here will facilitate future work relating these neural phenomena to the structure of song variation as well as to song maturity.

Finally, we note that the sCBG circuitry not only regulates song variability but also supports successful juvenile copying [47–49]. In addition to future work on song variation, the approach developed here may help elucidate the mechanisms of song improvement during sensorimotor learning. Adult zebra finches adapt their vocal output to avoid producing vocal variants that elicit experimental punishment [50–56]. The behavioral expression of these punishment-driven song adaptations initially depends on the sCBG, although these behavioral changes eventually become sCBG-independent [53,55,56]. This observation motivates the hypothesis that song improvements during juvenile sensorimotor learning also initially depend on sCBG premotor activity [57,58]. Future work can use the dynamics of song distribution models or predicted age values to quantify juvenile song learning and relate these behavioral refinements to sCBG activity. More broadly, our approach offers a strategy to quantitatively characterize learning as it is revealed by an animal’s changing behavior without experimenter-given goals or reward structures.

## Materials and methods

### Ethics statement

All data in this study were acquired under the animal use protocol “Cellular Mechanisms of Learning and Memory in the Avian Brain” (registry number: A172-20-08), which was approved by the Duke University Institutional Animal Care and Use Committee.

### Data collection

We raised 5 juvenile male zebra finches in their home cages where their father was the sole adult male and song model. To begin collecting song data, we isolated each juvenile in a sound box equipped with a microphone (recording onset ages given in S1 Table). We collected sound-triggered audio data at 44100 Hz with SAP [18] (3 birds) or at 32000 Hz with EvTaf [54] (2 birds). We recorded until birds reached at least 94dph (recording offset ages given in S1 Table).

### Syllable features

To extract the acoustic features of song syllables, we used a variational autoencoder [21–23], with a previously published training procedure and architecture [25]. This VAE encodes spectrogram images as a variational posterior in 32-dimensional latent space using a series of convolutional layers followed by a series of fully connected layers. Samples in latent space are decoded using a series of fully connected layers, followed by a series of transpose convolutions. We trained VAEs separately for each bird in our dataset. In brief, we hand-tuned sound amplitude-based segmentation parameters to extract all individual sounds from the entire developmental audio record of each animal. We saved a spectrogram corresponding to each sound. The spectrogram was the log modulus of the short-time Fourier transform of the audio waveform with 128 mel-spaced frequency bins. In order to keep VAE input size fixed, short sounds were symmetrically zero padded on the x (time) axis to a width of 128 pixels. Spectrogram floor and ceiling values were manually tuned to a range that captured variation in vocal sound intensity but excluded quiet background noise. These clipped syllable spectrograms were rescaled, so all values fell in the interval [0,1]. For each bird, we designated 30,000 random sound spectrograms as the bird-specific VAE training dataset. For the three birds collected using SAP [18] we trained the autoencoder for 500 epochs; for the two birds collected with EvTAF [54], we trained the autoencoder for 100 epochs after observing qualitatively successful reconstruction accuracy at that reduced training duration. Finally, the resulting trained VAEs were used to calculate a latent representation of every sound in each animal dataset, by calculating the mean of the latent variational posterior given by the trained VAE encoder.

For some benchmark analyses, we also calculated spectrogram latent features based on linear dimensionality reduction. After grouping syllable renditions based on clustering in VAE latent space (see next section), we performed within-syllable principal components analysis directly on syllable spectrograms. We represented syllables using the top principal components, truncating the representation at a principal component number equal to the effective dimensionality of the VAE latent space representation of that syllable.

### Syllable labeling

After calculating a latent representation for every sound in the dataset, the latents were embedded in a 2-dimensional UMAP to visualize clusters (UMAP run in python [31] with n_neighbors = 20, min_dist = 0.1, metric = ‘euclidean’). By investigating the underlying spectrograms of renditions in each cluster, we were able to assign meaningful category labels to different clusters. Some categories (like cage noise and call types) were discarded. We retained for further analysis only clear clusters corresponding to syllable types represented in the animal’s crystallized endpoint song. An example UMAP of labeled sounds is given in S1 Fig Finally, we performed within-syllable principal components analysis to find the primary axes of variation exhibited by syllables over the course of development.

### Predicted age network training

For each animal, we partitioned the entire collected repertoire of song syllables into a training set and an evaluation set (80/20 split). We trained predicted age networks (see Fig 2A), using the 32-dimensional latent vector describing each observed spectrogram as network input. The network minimized an error consisting of squared prediction error and a regularization term consisting of the sum of squares of the network weights. These terms were combined with weights given by Bayesian regularization [59] by training the network in MATLAB using the fitnet function (with parameter “trainFcn” = “trainbr”). We included regularization in order to “smooth” the predicted age function in acoustic space, to improve generalizability for a different set of experiments in which probe days of data were systematically withheld from training. The training data input to this procedure was automatically partitioned by MATLAB into a 70/15/15 split corresponding to training, test, and validation sets respectively. Training iterated until performance on the test set failed to improve for three consecutive epochs, or until 30 minutes had elapsed. The validation subdivision was unused, as we used the previously withheld evaluation partition to evaluate our trained models.

### Predicted age network evaluation

We quantified the performance of the predicted age networks relative to shuffle controls and alternative (linear, or shallower architecture) models as mean squared error (MSE) on our held-out evaluation datasets (see previous section). Evaluation was limited to renditions produced from 60 to 95dph. We compared network performance against two shuffle benchmarks. In the “total shuffle” benchmark, we shuffled production ages relative to latent descriptions for all syllables in the test set. Then we computed the MSE of model predictions based on shuffled ages. For each syllable, we performed this permutation test 1000 times and recorded the average MSE from these experiments as the “total shuffle” benchmark performance score. In the “within-day shuffle” benchmark, we again shuffled ages relative to latent vectors, but we prevented shuffles that swapped the ages of syllables produced on different days. As with the other benchmark, we performed 1000 “within-day shuffle” experiments and recorded the average MSE from these experiments as the “within-day shuffle” benchmark performance score. We compared performance against a linear prediction of age from latents, against a comparable network using syllable spectrogram principal components, and against a shallower network architecture (see S3 Fig).

### Analysis of overnight predicted age shifts

For every syllable, renditions were binned by age at production time, using a bin width of 0.1 days. We calculated the 1^st^, 5^th^, 25^th^, 50^th^, 75^th^, 95^th^, and 99^th^ percentile predicted age in each data bin, and discarded bins containing fewer than 30 syllable renditions. We subsequently calculated percentile-wise overnight shifts in predicted age by subtracting the ith predicted age percentile in the last time bin of day j from the ith predicted age percentile in the first time bin of day j+1. In the event that the last time bin on day j and the first time bin on day j+1 were separated by more than 0.6 days (which occurred in infrequent cases where morning or evening data was lost due to acquisition equipment errors), the overnight comparison was discarded.

We created linear mixed effects models of percentile-wise overnight shifts. We included fixed effects of intercept and percentile slope. The model included a bird-level random effects vector (bbird), populated with intercept-percentile slope pairs (b_0,I_,b_1,i_) for each bird i distributed according to the 2D Gaussian N(0,Θ) estimated from the data. Similarly, the model included a syllable-level random effects vector (bsyll), populated with intercept-percentile slope pairs (b_0,j_,b_1,j_) for each syllable j according to N (0, Φ) estimated from the data. The model was fit using MATLAB’s fitlme function and providing the Wilkinson notation formula:

$$“\text{overnight}\;\text{shift}∼1+\text{percentile}+\left(1\;+\text{percentile}|\text{bird}\right)+\left(1\;+\text{percentile}|\text{syllable}\right).”$$

We repeated this data filtering and modeling procedure for alternative percentile binning decisions. In particular, we alternatively used 3 percentile bins (at the 1^st^, 50^th^, and 99^th^ percentile level), and 11 percentile bins (at the 1^st^, 5^th^, 15^th^, 25^th^, 40^th^, 50^th^, 60^th^, 75^th^, 85^th^, 95^th^, and 99^th^ percentile level). Fixed effects coefficients in S2 Table.

### Gaussian model training

Gaussian models were trained to fit data separately for different syllable types, using the same 80% training (vs 20% evaluation) dataset split used to divide data for predicted age network training and evaluation. Observations consisted of pairs: production time and a vector specifying latent location. Rather than using full-rank covariance matrices, we modeled only the number of principal components needed to explain >99% of the syllable’s latent space variation (6 to 8 principal components). The training data was further subdivided into train and test sets according to a 80/20 split. These were used for weight updating and training termination, respectively. The architecture for these Gaussian model networks is given in Fig 4A. The model input “age” was generated from observed data by z-scoring all production ages. The sin(time) and cos(time) inputs were generated from production time of day by calculating the sine and cosine, respectively, of production time of day in radians (24 hrs = 2π). Thus, the quantity [sin(time), cos(time)] took unique values at every time of day, and identical values for renditions at the same time of day on different days.

The output values of the network were interpreted as three distinct vectors (μ,l, and d; see Fig 4B). We used these vectors to construct the parameters of a multivariate Gaussian distribution with full covariance matrix Σ of dimension k (with k varying between 6 and 8 on a syllable-type basis according to the number of principal components preserved, as described above). Output l is reshaped into a lower triangular matrix L reflecting the Cholesky decomposition of Σ: Σ = LL^T^ + diag(e^d^ + ε). μ is directly interpreted as the multivariate Gaussian mean. Given training examples of paired production times and latent locations, the network learned to minimize the negative log likelihood given latent observations: $\sum_{i=1}^{n}-\text{log}\left(P({latent}_{i})\right),P\sim N\left(\mu ,\Sigma |{age}_{i}\right)$ over n samples. To minimize this loss with respect to the network parameters, we used the Adam optimizer with learning rate = 0.001. The model was trained until performance on the test set stopped improving, at which point the model producing the best test set performance was saved and subsequently used in analysis. The same data partitioning and training procedure was used for the alternative architectures in S4A and S4B Fig, and similarly for the Gaussian models in PCA latent space.

### Evaluation of Gaussian models

We scored the performance of the trained Gaussian models using held out syllable rendition data. First, we calculated the log likelihood of the trained model by evaluating $\frac{1}{n}\sum_{i=1}^{n}\text{log}(P({latent}_{i}|{age}_{i}))$ on the n held-out evaluation renditions (20% of all renditions for each syllable). To evaluate the contribution of age information to the performance of each model, we generated 1000 permutations of the age parameter and computed for each permutation the “total shuffle” log likelihood $\frac{1}{n}\sum_{i=1}^{n}\text{log}(P({latent}_{i}|{age}_{total\;shuffle\;index}))$. We summarized this experiment for each syllable with the mean of the total shuffle log likelihood. To separate the contribution of within- and between-day age information to the performance of each model, we generated 1000 within-day permutations of the age parameter. That is, permutations that associated latent observations with wrong-day production times were prohibited. For each permutation we computed the “within-day shuffle” log likelihood $\frac{1}{n}\sum_{i=1}^{n}\text{log}(P({latent}_{i}|{age}_{within-day\;shuffle\;index}))$. We summarized this experiment for each syllable with the mean of the within-day shuffle log likelihood.

### Entropy analysis

The trained Gaussian models map production ages to multivariate Gaussian distributions. The volume of these distributions is summarized by their entropy: $\frac{1}{2}\text{ln}\;\left|\Sigma \right|\;+\;\frac{k}{2}(1\;+\;\text{ln}\;2\pi )$, where k is the number of dimensions. In order to evaluate the behavior of the fitted models we generated “query times” at 5-minute intervals during daytime hours of birds’ light cycles. Because the model output is not reliable at times without nearby training data, we then discarded query times at which fewer than 30 training renditions of the modeled syllable were produced in the half hour window centered at the query time. At the remaining query times, we generated predicted covariance matrices and calculated entropy from these.

The number of principal components, k, used to represent syllable rendition acoustics differed across syllables. In general, entropy scales with distribution dimension, so the differences in k between syllables almost certainly leads to variation in entropy that has no relation to production time. More generally, to account for variation in entropy that correlated with bird or syllable identity, we constructed a linear mixed effects model for the dependence of entropy on time of day. We included fixed effects of intercept and time of day. The model included a bird-level random effects vector (bbird), populated with intercept and slope (vs. time of day) pairs (b_0,i_,b_1,i_) for each bird i distributed according to the 2D Gaussian N (0, Θ) estimated from the data. Similarly, the model included a syllable-level random effects vector (bsyll), populated with intercept and slope pairs (b_0,j_,b_1,j_) for each syllable j according to N (0, Φ) estimated from the data. The model was fit using MATLAB’s fitlme function and providing the Wilkinson notation formula:

$$“\text{entropy}∼1+\text{timeOfDay}+\left(1+\text{timeOfDay}|\text{bird}\right)+\left(1+\text{timeOfDay}|\text{syllable}\right).”$$

We used the same sampling and modeling procedure to describe the daily entropy trajectories of the alternative model architectures in S4 Fig. We used a similar model to evaluate entropy trends in PCA-based models.

### Simulations of development

For every sample age in our held-out evaluation datasets, we calculated a Gaussian distribution from the corresponding trained Gaussian model and sampled from it once. The syllable-level collections of these samples constituted our “baseline” simulated developmental datasets. To sample from “fixed-entropy” models, we first calculated a target covariance determinant: the minimum |Σ| for each syllable on each day (the log of this value is proportional to the minimum entropy achieved). For each sample age in our held-out test datasets, we calculated a Gaussian distribution from the corresponding, trained model. We factored the sample model’s covariance matrix into an eigenvector matrix Q and eigenvalue matrix Λ: Σ = QΛQ^T^. We sought to normalize the product of the eigenvalues so that this product equaled the target covariance determinant. To do this, we calculated a relevant “normalization ratio” relating the current and target covariance determinants: Norm. ratio = $\frac{{\left|\Sigma \right|}_{current}}{{\left|\Sigma \right|}_{target}}$. We then divided each eigenvalue in Λ by $\sqrt[{k}]{norm\;ratio}$ where k is the total number of dimensions of the Gaussian model: $\Lambda_{scaled}=\sqrt[{k}]{norm\;ratio}\Lambda$. We reconstructed a fixed-entropy model covariance matrix from the eigenvector matrix and normalized eigenvalue matrix as: Σ_*fixed entropy*_ = *Q*Λ_*scaled*_Q^*T*^. We sampled fixed entropy models in the same way as our baseline models to generate our “fixed-entropy” simulation datasets.

We calculated percentile-wise overnight shifts from our simulations through the procedure described above (Analysis of Overnight Predicted Age Shifts). We used a linear mixed effects model to quantify the effects of quantile and entropy condition on overnight shifts in our simulation, while incorporating random effects in all the fitted coefficients at the bird and syllable level, similar to the mixed effects models described earlier. This analysis was performed in MATLAB using the fitlme function and Wilkinson notation:

$$“\text{shift}\sim 1+\text{quantile}*\text{entropy}\_\text{condition}+\left(1+\text{quantile}*\text{entropy}\_\text{condition}|\text{bird}\right)+\left(1+\text{quantile}*\text{entropy}\_\text{condition}|\text{syllable}\right).”$$

We repeated this analysis of simulated data using different quantile binning strategies and after simulations using different Gaussian model architectures.

## Supporting information

S1 Fig Identification and labeling of syllables. Scatterplot of sounds in UMAP of latent space following label assignment. Note that short calls, short cage noise sounds, and intro notes could be partially overlapping, but song syllables were readily distinguishable from one another and from all other sounds.
(TIF)

S2 Fig Developmental spectrogram reconstruction. Averages (top rows) of 50 random spectrograms and reconstruction (bottom rows) of corresponding renditions’ mean latent location. Columns (left to right) are generated by sampling renditions produced on 63dph, 73dph, 83dph, and 93dph.
(TIF)

S3 Fig Alternative age prediction approaches. **A.** Architecture of comparison predicted age network with fewer layers. **B.** Prediction method significantly impacts results (within-syllable anova, n = 13 syllables, F(3,36) = 59.542,p = 4.9493*10^−14^). Mean squared error was lower for our original model than total shuffle control (shuffle-original = 94.857, p = 3.1453*10^−5^, and see main text). MSE was also lower for our original model than linear age predictions from VAE latents (linear-original = 9.5655, p = 0.010971). However, there was no significant difference in MSE between our original model and a shallower model with 601 trained parameters (p = 0.81227). **C** Performance of predictive network based on VAE latents versus spectrogram principal components. We trained a network to predict age from a PC subspace of spectrogram space with the same effective dimensionality as our VAE latent space (i.e., the dimensionality at which we modeled syllable distributions over time). For 12 of 13 syllables, predictions based on VAE latent representations outperformed predictions based on linear (PCA) components. Despite an outlying large error for the VAE-based prediction for one syllable, the MSE reduction in VAE-based models vs PCA-based models is statistically trending in a syllable-level repeated measures ANOVA (PCA-VAE MSE = 7.206, p = 0.073).
(TIF)

S4 Fig Multiple architectures for inferring syllable distributions. **A-B**. We tested a network with 64 neurons per layer **(A)** and a network with reduced number of layers (**B**). **C**. These networks exhibited qualitatively similar performance on unshuffled data (none), as well as total (total) and within-day (wd) shuffled data. Lines connect measures from individual syllables, and markers reflect bird identity.
(TIF)

S1 Table Counts and age ranges of different syllables recorded in this study. Note that predicted age and Gaussian model training was performed on random subsets of these syllables, but downstream analysis using trained models was limited to renditions produced between 60 and 95 dph.
(XLSX)

S2 Table Quantile binning and overnight maturity shifts. Fixed effects parameter estimates (+/- standard errors) for models of overnight maturity shifts by quantile, for different quantile binning strategies. Across binning strategies, the overnight shift depends significantly on quantile value. All models included random effects in the intercept and slope at the bird (n = 5) and syllable (n = 13) level.
(XLSX)

S3 Table Multiple Gaussian model architectures exhibit circadian entropy change. Parameter estimates (+/- standard errors) for models of syllable distribution entropy by time of day, using different network architectures to estimate syllable distributions. Across architectures, entropy decreased significantly with time of day by similar amounts. All models included random effects in the intercept and slope at the bird (n = 5) and syllable (n = 13) level.
(XLSX)

S4 Table Quantile binning and overnight shifts of simulated data. Fixed effects parameter estimates (+/- standard errors) for comparisons of quantile-dependent overnight shifts in performance in simulations with free or fixed within-day entropy. The quantile-dependence of overnight shifts is significant in free-entropy simulations and eliminated by a significant interaction with entropy condition in fixed-entropy simulations, regardless of the quantile binning strategy. We included random effects of bird (n = 5) and syllable (n = 13) for all parameters.
(XLSX)

S5 Table Gaussian model architecture and overnight shifts of simulated data. Fixed effects parameter estimates (+/− standard errors) for comparisons of quantile-dependent overnight shifts in performance in simulations with free or fixed within-day entropy. We used different Gaussian model architectures to simulate development. The quantile-dependence of overnight shifts is significant in free-entropy simulations and eliminated by a significant interaction with entropy condition in fixed-entropy simulations, regardless of Gaussian model architecture. We included random effects of bird (n = 5) and syllable (n = 13) for all parameters.
(XLSX)
