# Supplementary figures and images for: Generative models of birdsong learning link circadian fluctuations in song variability to changes in performance
Source: PLoS Comput Biol. 2023 May 1;19(5):e1011051. doi: 10.1371/journal.pcbi.1011051 (PMC10150982; doi:10.1371/journal.pcbi.1011051)

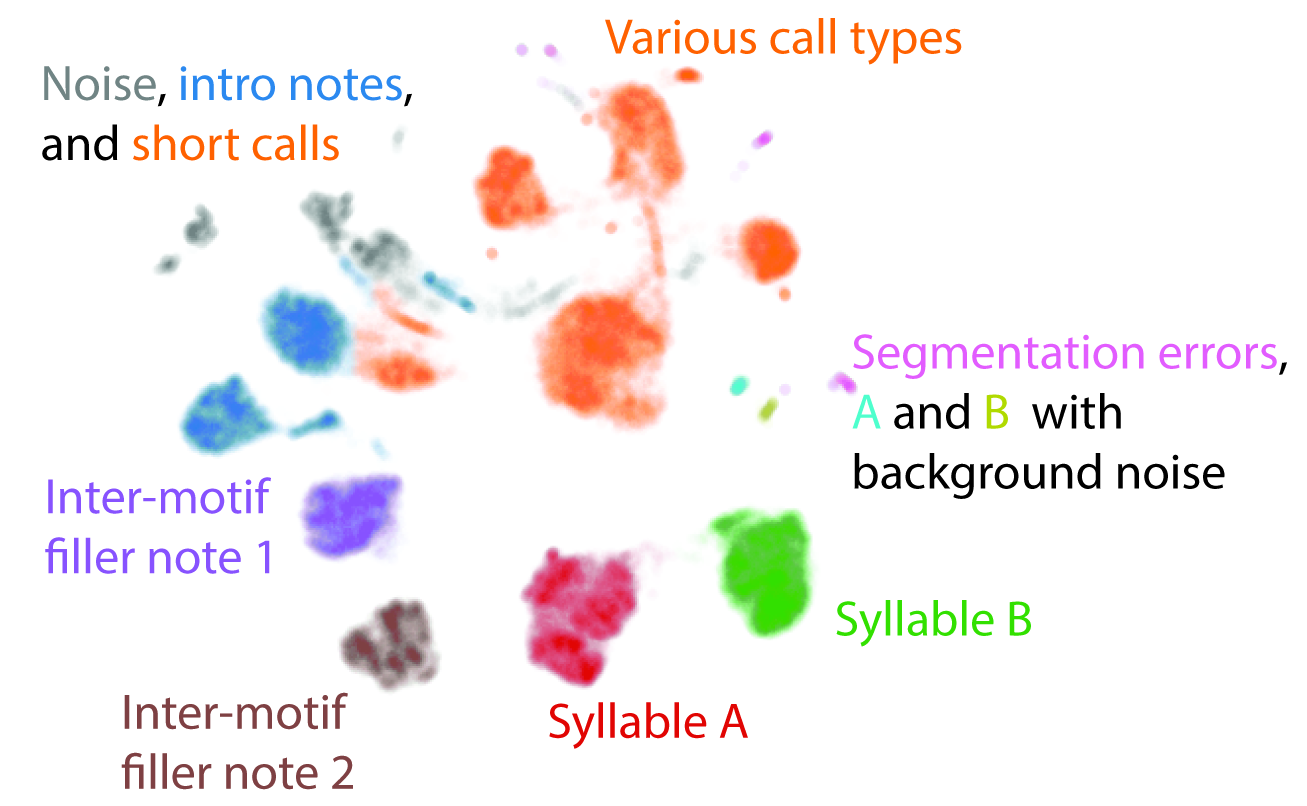

Supplement: S1 Fig — Scatterplot of sounds in UMAP of latent space following label assignment. Note that short calls, short cage noise sounds, and intro notes could be partially overlapping, but song syllables were readily distinguishable from one another and from all other sounds. (TIF) [file pcbi.1011051.s001.tif]

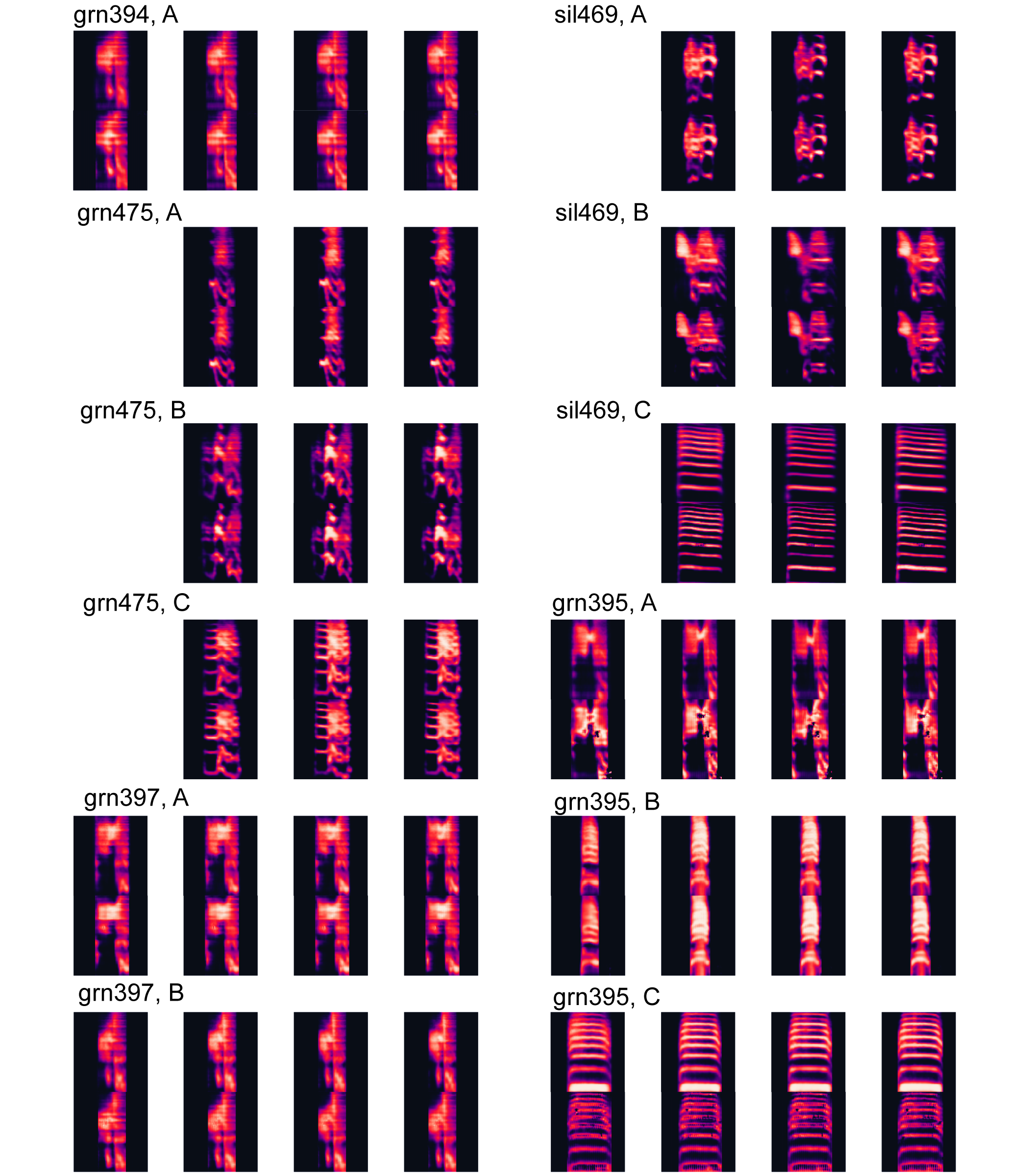

Supplement: S2 Fig — Averages (top rows) of 50 random spectrograms and reconstruction (bottom rows) of corresponding renditions’ mean latent location. Columns (left to right) are generated by sampling renditions produced on 63dph, 73dph, 83dph, and 93dph. (TIF) [file pcbi.1011051.s002.tif]

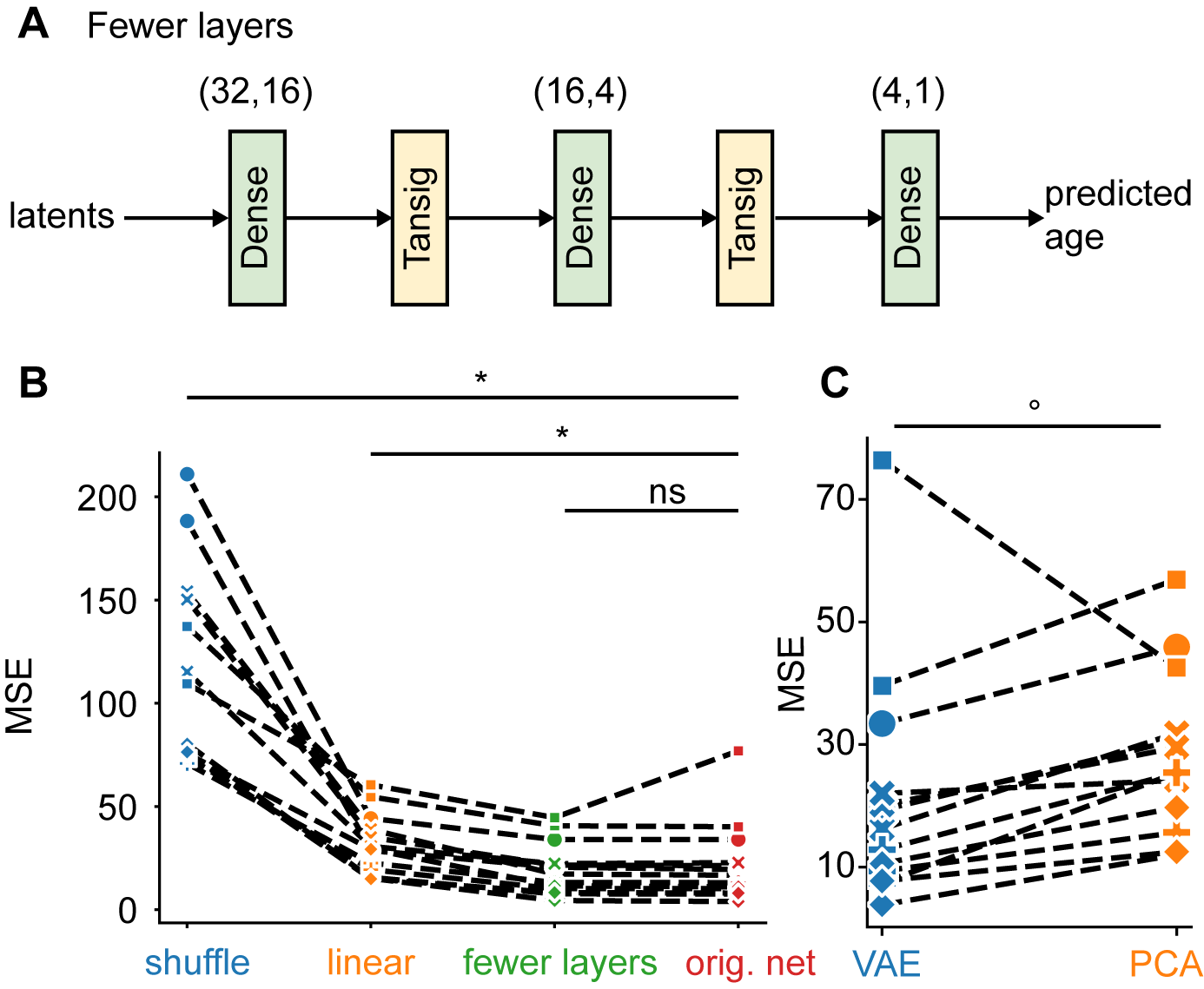

Supplement: S3 Fig — A. Architecture of comparison predicted age network with fewer layers. B. Prediction method significantly impacts results (within-syllable anova, n = 13 syllables, F(3,36) = 59.542,p = 4.9493*10−14). Mean squared error was lower for our original model than total shuffle control (shuffle-original = 94.857, p = 3.1453*10−5, and see main text). MSE was also lower for our original model than linear age predictions from VAE latents (linear-original = 9.5655, p = 0.010971). However, there was no significant difference in MSE between our original model and a shallower model with 601 trained parameters (p = 0.81227). C Performance of predictive network based on VAE latents versus spectrogram principal components. We trained a network to predict age from a PC subspace of spectrogram space with the same effective dimensionality as our VAE latent space (i.e., the dimensionality at which we modeled syllable distributions over time). For 12 of 13 syllables, predictions based on VAE latent representations outperformed predictions based on linear (PCA) components. Despite an outlying large error for the VAE-based prediction for one syllable, the MSE reduction in VAE-based models vs PCA-based models is statistically trending in a syllable-level repeated measures ANOVA (PCA-VAE MSE = 7.206, p = 0.073). (TIF) [file pcbi.1011051.s003.tif]

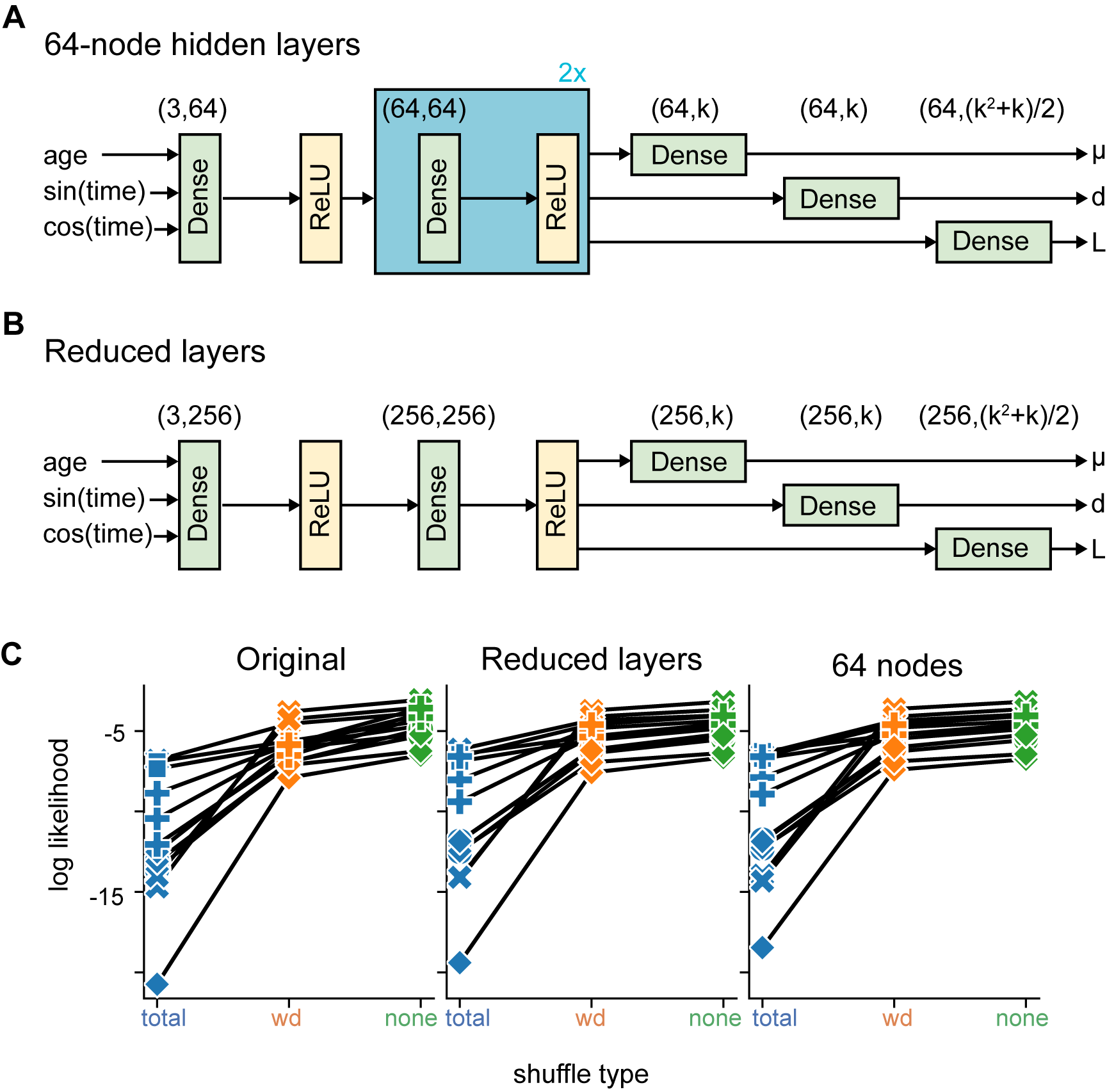

Supplement: S4 Fig — A-B. We tested a network with 64 neurons per layer (A) and a network with reduced number of layers (B). C. These networks exhibited qualitatively similar performance on unshuffled data (none), as well as total (total) and within-day (wd) shuffled data. Lines connect measures from individual syllables, and markers reflect bird identity. (TIF) [file pcbi.1011051.s004.tif]
